# Water Hyacinth Fiber as a Bio-Based Carbon Source for Intumescent Flame-Retardant Poly (Butylene Succinate) Composites

**DOI:** 10.3390/polym15214211

**Published:** 2023-10-24

**Authors:** Anothai Suwanniroj, Nitinat Suppakarn

**Affiliations:** 1 School of Polymer Engineering, Institute of Engineering, Suranaree University of Technology, Nakhon Ratchasima 30000, Thailand; 2 Center of Excellence on Petrochemical and Materials Technology, Chulalongkorn University, Bangkok 10330, Thailand; 3 Research Center for Biocomposite Materials for Medical Industry and Agricultural and Food Industry, Suranaree University of Technology, Nakhon Ratchasima 30000, Thailand

**Keywords:** poly (butylene succinate), water hyacinth, ammonium polyphosphate, intumescent flame retardant, flame retardancy

## Abstract

In this study, flame-retardant poly (butylene succinate) (PBS) composites were developed utilizing a bio-based intumescent flame retardant (IFR) system. Water hyacinth fiber (WHF) was used as a bio-based carbon source, while ammonium polyphosphate (APP) served as both an acid source and a blowing agent. Effects of WHF:APP weight ratio and total IFR content on the thermal stability and flammability of WHF/APP/PBS composites were investigated. The results demonstrated that the 15WHF/30APP/PBS composite with a WHF to APP ratio of 1:2 and a total IFR content of 45 wt% had a maximum limiting oxygen index (LOI) value of 28.8% and acquired good flame retardancy, with a UL-94 V-0 rating without polymer-melt dripping. Additionally, its peak heat release rate (pHRR) and total heat release (THR) were, respectively, 53% and 42% lower than those of the neat PBS. Char residue analysis revealed that the optimal WHF:APP ratio and total IFR content promoted the formation of a high graphitized intumescent char with a continuous and dense structure. In comparison to the neat PBS, the tensile modulus of the 15WHF/30APP/PBS composite increased by 163%. Findings suggested the possibility of employing WHF, a natural fiber, as an alternative carbon source for intumescent flame-retardant PBS composites.

## 1. Introduction

Biodegradable environmentally friendly polymers have recently emerged as a possible long-term solution for the reduction in the dependency of the industrial sector on petroleum-based plastics [1,2]. Poly (butylene succinate) (PBS), a biodegradable aliphatic polyester derived from the polycondensation of succinic acid and butanediol, is one of the most competitive biodegradable polymers, and particularly attractive due to its superior biodegradability, processability, and thermal stability [3,4]. The tensile strength and elongation at break of PBS are comparable to polyethylene (PE) and polypropylene (PP), while the stiffness of PBS falls between low-density polyethylene (LDPE) and high-density poly ethylene (HDPE) [3]. However, PBS, like other thermoplastic resins, is naturally flammable, possessing a limiting oxygen index (LOI) value of around 23.3%. During combustion, PBS also releases substantial amounts of polymer melt droplets, which ignite cotton placed beneath the specimen and subsequently promote the spread of the fire [5]. This severely limits the potential applications of PBS, particularly in foaming products, packaging, and electronic industries. Therefore, reducing PBS flammability is a challenge for future applications requiring high flame retardancy.

The addition of flame-retardant chemicals to PBS is a cost-effective approach to reducing its flammability and minimizing the risk of fire. According to the fire triangle, three components are required for fire to occur: oxygen, heat, and combustible material or fuel. Therefore, to prevent PBS combustion, heat or oxygen must be removed. Flame retardants function chemically and/or physically in the condensed or gaseous phases by either increasing the ignition resistance of PBS or slowing the rate of flame spread [6,7]. Many flame retardants have been investigated for their efficiency in enhancing the flame retardancy of PBS. These included inorganic (nano) particles such as magnesium hydroxide [8], carbon black [9], molybdenum disulfide [10], expandable graphite [11], nano clay [12], lignin [13,14,15], and intumescent flame-retardant systems [16,17].

Intumescent flame-retardant (IFR) systems have been extensively utilized to reduce the flammability and melt-dripping of polymers. They provide considerable advantages over traditional flame retardants such as halogenated flame retardants and metallic hydroxides due to their low toxicity and high flame-retardant efficiency [18,19]. A conventional IFR system consists of three main components as an acid source, a carbonizing agent, and a blowing agent. When a material containing IFR is exposed to heat or flame, it expands and forms a swollen carbonaceous layer that acts as a physical barrier to stop heat and pyrolysis byproducts from immediately penetrating the surface of the material [18]. The decomposition of IFR compounds results in the emission of non-flammable gases, such as the production of ammonia gas and water during the decomposition of APP. The released incombustible ammonia gas dilutes the concentration of oxygen and fuel in the gaseous phases, slowing the combustion and decreasing heat release. Several research groups have explored the utilization of intumescent flame retardants in PBS. Kuan et al. used the water crosslinking reaction to improve the interface between APP and PBS. As a result, the PBS composite with 15% APP was non-dripping and had a UL-94 V-0 flame retardancy rating [16]. Yue et al. formulated a lignin chelate as a catalyst for use with IFR chemicals [20]. They found that replacing IFR additives with 2% lignin chelate enhanced the mechanical properties and flame retardancy performance of IFR/PBS composites. Gu et al. synthesized urea-intercalated kaolinite (U-Kaol) as a synergistic flame retardant for PBS [21]. After adding 5 wt% U-Kaol and 20 wt% IFR, the PBS composite passed the UL-94 V-0 rating, and its LOI increased from 21.9% to 40%. Chen et al. found that the addition of fumed silica, APP, and MEL in the appropriate proportions considerably enhanced the flame retardancy and anti-dripping properties of PBS [22].

A conventional IFR system is a combination of ammonium polyphosphate (APP) and pentaerythritol (PER). APP serves as both an acid source and a blowing agent, while PER is used as a carbon source. These chemicals play an important role in the formation of carbonaceous char. The optimal weight ratio of an acid source to a charring agent (APP: PER) produces a strong expanded carbon layer with a certain hole size, which enhances the flame-retardant performance of the material [23,24]. APP is a commonly used and ecologically safe flame retardant that produces phosphoric acid and releases ammonia gas during combustion. PER is a polyhydroxy compound derived from petrochemistry. However, due to its high energy consumption during production and significant pollutant emissions after burning, PER is incompatible with sustainable development standards [25]. Moreover, it has low charring performance and exhibits deteriorated char-forming ability, particularly when used in biodegradable polyesters [26].

To address environmental concerns and enhance the performance of intumescent flame retardants (IFRs), researchers have been exploring the use of eco-friendly carbon sources combined with ammonium polyphosphate (APP). In recent years, several bio-based substances with unique polyhydroxy structures and char-forming abilities have been investigated, including lignin [27], starch [28], cyclodextrin [29], chitin [30], kenaf [31], bamboo [32], and soy protein [33]. Among these, water hyacinth has emerged as a promising bio-based carbon source.

Water hyacinth, a free-floating aquatic plant known for its rapid growth and negative impact on ecosystems, is composed of cellulose (20%), hemicellulose (33%), and lignin (9%), with numerous hydroxyl groups [34]. Due to its chemical composition, water hyacinth fiber (WHF) can be used to strengthen polymer composites [5,35]. It also has the potential to substitute conventional carbon sources in IFR systems.

In our previous study, we demonstrated that incorporating poly (butylene succinate) grafted with glycidyl methacrylate (PBS-*g*-GMA) into WHF/APP/PBS composites improves their mechanical and flame-retardant properties [5]. However, the weight ratio of WHF (a carbon source) to APP (an acid source) and the overall IFR content have not been explored in relation to the flame retardancy performance of WHF/APP/PBS composites.

Considering the significant influence of the acid source to char ratio on IFR system performance, several studies have investigated the enhancement of flame retardancy by optimizing the weight ratio between APP and different carbon sources. For instance, Wang et al. developed a biodegradable intumescent flame retardant using soy protein (SP) as a char and gas source in combination with APP for PBS matrices [33]. Their findings revealed that a PBS composite with an APP:SP weight ratio of 2:1 exhibited excellent char-forming ability, a notable increase in limiting the oxygen index (LOI) value, a decrease in peak heat release rate (pHRR), and an increase in total smoke release (TSR). Hu et al. used poly(isosorbide carbonate) (PIC), a bio-based polymer, as a green char-forming agent in PBS/APP composites [36]. When 7.5 wt% of PIC was added to PBS/APP, the pHRR and THR decreased by 24% and 48%, respectively while the residue yield increased to 44%. Yue et al. prepared flame-retardant modified casava dregs-PBS composites with different amounts of IFR [26]. At total filler content of 30 wt% and ratio of cassava dregs to IFR 1:5, PBS composites achieved a UL-94 V-0 rating with an LOI value of 37.3%.

This study developed flame-retardant PBS composites utilizing a green IFR system. WHF was substituted for PER as a bio-based carbon source, while APP served as both an acid source and a blowing agent. Effects of WHF:APP weight ratio and total IFR content were investigated to assess the thermal stability, flame retardancy, and tensile properties of WHF/APP/PBS composites.

## 2. Materials and Methods

### 2.1. Materials

Poly (butylene succinate) (PBS) (BioPBSTM Fz71PM) with melt flow index (MFI) of 22.0 g/10 min (190 °C, 2.16 kg) and density of 1.26 g/cm^3^ was purchased from PTT MCC Biochem Co., Ltd., Rayong, Thailand. Water hyacinth fiber (WHF) was gathered from the Chao Phraya River, Pathum Thani, Thailand, ground using a wood-crushing machine, and sieved to particle size range 106 to 212 µm. Ammonium polyphosphate (APP) was obtained from JLS Flame Retardants Chemical Co., Ltd., Hangzhou, China. Glycidyl methacrylate-grafted poly (butylene succinate) (PBS-*g*-GMA) was used as compatibilizer for PBS composites. It was synthesized in our laboratory by melt blending of 3 phr of dicumly peroxide (DCP) and 0.5 phr of glycidyl methacrylate (GMA) [5].

### 2.2. Methods

#### 2.2.1. Preparation of WHF/APP/PBS Composites

Before processing, WHF and APP were dried for 12 h at 60 °C, while PBS and PBS-*g*-GMA were dried for 3 h at 80 °C in a vacuum oven. Then, PBS, APP, WHF, and PBS-*g*-GMA were melt-mixed at 135 °C for 10 min in a Rheomix 3000 internal mixer (Haake, Vreden, Germany) with 60 rpm rotor speed. Weight ratio of WHF:APP and total weight content of WHF and APP (IFR components) were varied. Formulations and designations of WHF/APP/PBS composites are shown in Table 1. After mixing, the samples were compressed into test specimens using a compression molding machine (Chareon Tut, Samutprakarn, Thailand) at 135 °C for 10 min under a pressure of 10 MPa and then cooled to room temperature.

#### 2.2.2. Determination of Flame Retardancy of WHF/APP/PBS Composites

UL-94 vertical burning tests were carried out in accordance with the ASTM D3801 standard [37] using a horizontal vertical flame chamber instrument (Atlas Material Testing Technology, Mount Prospect, IL, USA). The dimensions of the specimens were 130 mm × 13 mm × 3 mm. The UL-94 test classifies materials by assigning them a burning rating, i.e., V-0, V-1, and V-2, based on their burning and afterglow times and dripping behaviors. For each formulation, at least five specimens were examined.

UL-94 horizontal burning tests were conducted according to ASTM D635 [38]. The test specimens had dimensions of 130 mm × 13 mm × 3 mm. The horizontal burning test provides a burning rate (V) of a specimen. V = 60 L/t where L is the burnt distance in mm, and the duration of burning t is measured in seconds.

Limiting oxygen index (LOI) tests were measured using a limiting oxygen index tester (Stanton Redcroft Ltd., London, UK) according to ASTM D2863 [39]. The dimensions of the LOI test specimens were 100 mm × 6.5 mm × 3 mm. Each formulation was evaluated using at least three specimens.

#### 2.2.3. Determination of Melt Flow Index of WHF/APP/PBS Composites

Melt flow index (MFI) of PBS and PBS composites was determined in accordance with ASTM D128 [40] using a melt flow index tester (Dynisco, Morgantown, PA, USA) at 150–210 °C and 0.276 kg of load. A minimum of ten samples were collected and weighted to determine the mean values.

#### 2.2.4. Determination of Thermal Stability of WHF/APP/PBS Composites

Analyses of the thermal properties and char-forming abilities of PBS and PBS composites were carried out using a TGA/DSC 1 thermal gravimetric analyzer (Mettler Toledo, Greifensee, Switzerland). Each sample (10 mg) was placed in a platinum crucible and heated at 10 °C/min from 35 to 800 °C in nitrogen atmosphere.

#### 2.2.5. Determination of Combustion Behavior of WHF/APP/PBS Composites

Combustion characteristics of PBS and WHF/APP/PBS composites were examined according to ISO 5660 [41] using an i-cone mini cone calorimeter (Fire Testing Technology, West Sussex, UK). The 100 × 100 × 3 mm specimens were wrapped in aluminum foil and subjected to a 35 kW/m^2^ heat.

#### 2.2.6. Determination of Morphologies and Structure of Char Layer

Following testing in a cone calorimeter, morphologies of the char layer were observed using a JSM 7800F field emission scanning electron microscope (FE-SEM) (JEOL Inc., Peabody, MA, USA) at 15 kV acceleration voltage.

Graphitic structures of char residues after the cone calorimetry test were investigated using an FT-Raman spectrometer (Bruker, Billerica, MA, USA) at room temperature. A sample was exposed to a 532 nm helium-neon laser for 3 s with a typical laser power of 12 mW. Its Raman spectrum was acquired in a scanning range of 50–4450 cm^−1^ with a resolution of 16 cm^−1^.

#### 2.2.7. Determination of Evolved Products during Thermal Decomposition of WHF/APP/PBS Composites

Evolved products from WHF/APP/PBS composites decomposing at their highest rate were determined using a TGA4000 thermal gravimetric analyzer (PerkinElmer, Waltham, MA, USA) directly coupled to a Spotlight200i Fourier transform infrared spectrometer (PerkinElmer, Waltham, MA, USA). The experimental setup for each instrument was described in the previous sections.

## 3. Results and Discussion

### 3.1. Flame Retardancy of WHF/APP/PBS Composites

**Figure 1:**
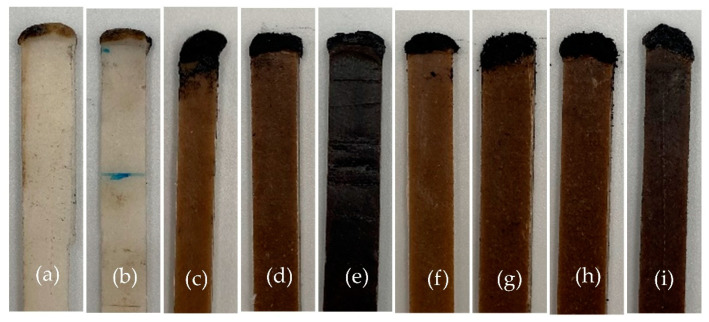
Digital photographs of specimens after LOI test; PBS (**a**), 30APP/PBS (**b**), 30WHF/PBS (**c**), 9WHF/36APP/PBS (**d**), 11.3WHF/33.7APP/PBS (**e**), 15WHF/30APP/PBS (**f**), 13WHF/27APP/PBS (**g**), 12WHF/23APP/PBS (**h**), and 10WHF/20APP/PBS (**i**).

The flame retardancy of PBS and WHF/APP/PBS composites was investigated using LOI and UL-94 tests. The flame test results are shown in Table 2, with digital photographs of PBS and PBS composites after the LOI test shown in Figure 1.

Neat PBS had an LOI of 23.3% and a horizontal burning rate of 16.39 mm/min. During the vertical burning test, PBS demonstrated extensive melt-dripping of flaming material, which ignited cotton located beneath the specimen. As a result, PBS was rated as a UL-94 unclassified (NC). After the addition of 30 wt% APP, the 30APP/PBS composite had an LOI of 32.0% and achieved a UL-94 V-0 rating. However, the dripping tendency of its molten state was still visible. After the addition of 30 wt% WHF, the 30WHF/PBS composite had an LOI of 20.6% and received a UL-94 NC rating, indicating the adverse impact of WHF on the flame retardancy of PBS. These findings suggested that adding just one filler, WHF or APP, to PBS was insufficient to enhance PBS flame retardancy.

When the overall amount of IFR was fixed at 45 wt%, all WHF/APP/PBS composites achieved a UL-94 V-0 rating without any molten components dripping. They were also self-extinguishing, so their horizontal burning rate could not be monitored. The LOI of WHF/APP/PBS composites increased when the weight ratio of WHF to APP changed from 1:4 to 1:2. The 15WHF/30APP/PBS composite with a WHF:APP ratio of 1:2 yielded the highest LOI of 28.8%, 5.5 units higher than the neat PBS.

When the WHF:APP ratio was set at 1:2, the LOI value of WHF/APP/PBS composites decreased as overall IFR content decreased. Among these, the 10WHF/20APP/PBS composite with total IFR weight content of 30 wt% had the lowest LOI value of 23.7%. In addition, all WHF/APP/PBS composites with total IFR content less than 45 wt% received a UL-94 V-2 rating and exhibited extensive polymer melt-dripping. 

Flame test results showed that the total weight of IFR and the ratio of WHF to APP had significant influence on flame-retardant properties of PBS composites. The 15WHF/30APP/PBS composite with a WHF:APP ratio of 1:2 and total IFR weight of 45 wt% exhibited the highest flame retardancy. In contrast, the 10WHF/20APP/PBS composite with a WHF:APP ratio of 1:2 and total IFR weight of 30 wt% showed poor flame resistance.

### 3.2. Melt Flow Index (MFI) of WHF/APP/PBS Composites

**Figure 2:**
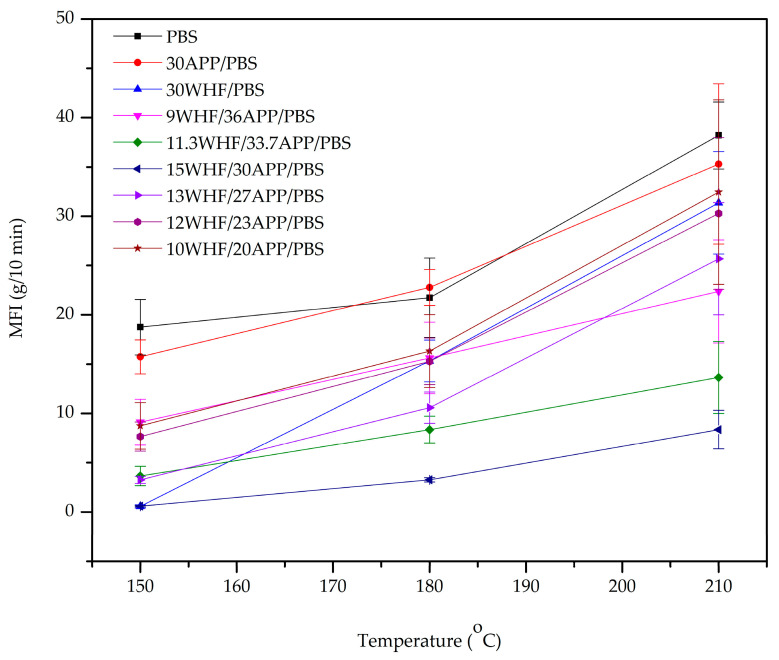
MFI values of neat PBS and WHF/APP/PBS composites.

The melt flow index (MFI) test is used to determine the flowability of a thermoplastic polymer during its molten state. The MFI value can be used to indirectly assess the effect of WHF and APP on anti-dripping properties, which enable the material to retain its shape during a fire. To prevent the decomposition of WHF and APP during testing, MFI measurements were performed at temperatures ranging from 150 to 210 °C. The relationships between the MFI values of PBS and PBS composites and temperature are shown in Figure 2.

As illustrated in Figure 2, the MFI values of all PBS composites were lower than neat PBS, suggesting that the addition of WHF and/or APP restricted the flowability of the PBS matrix. When either WHF or APP was added at 30 wt%, the MFI value of the 30WHF/PBS composite was lower than that of the 30APP/PBS composite. These findings supported the UL-94 flame results in that the anti-dripping abilities of the PBS matrix were not improved by simply adding WHF or APP.

When WHF and APP were combined as components of an IFR system, the MFI values of all WHF/APP/PBS composites decreased compared to neat PBS. These indicated an increase in viscosity of the molten PBS composite. When the overall IFR weight was fixed at 45 wt%, the MFI values of the PBS composites decreased as the WHF:APP ratio changed from 1:4 to 1:2. The results revealed that the addition of WHF at a constant IFR content increased the viscosity of the PBS composites. When the WHF:APP ratio was 1:2, the MFI values of the PBS composites increased as the total IFR weight decreased from 45 wt% to 30 wt%. These indicated that, at a constant WHF:APP ratio, the viscosity of PBS composites increased with increasing total IFR content.

The 15WHF/30APP/PBS composite with a WHF:APP ratio of 1:2 and total IFR weight of 45 wt% exhibited the lowest MFI value among the WHF/APP/PBS composites, while the 10WHF/20APP/PBS composite with a WHF:APP ratio of 1:2 and total IFR weight of 30 wt% showed the highest MFI value. These results provided insight on why the 10WHF/20APP/PBS composite exhibited significant polymer melt-dripping during the UL-94 flame test, whereas the 15WHF/30APP/PBS composite had no melt-dripping.

### 3.3. Thermal Stability of WHF/APP/PBS Composites

**Figure 3:**
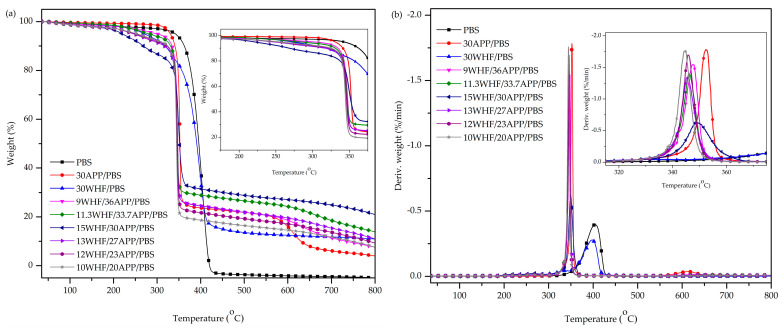
TGA (**a**) and DTG (**b**) thermograms of PBS and PBS composites.

The thermal properties and char-forming abilities of PBS and PBS composites were analyzed in a nitrogen atmosphere using a thermogravimetric analyzer (TGA). TGA and derivative (DTG) thermograms of the samples are shown in Figure 3a,b, respectively. Thermal parameters, i.e., T_5%_ (decomposition temperature at 5 wt% weight loss), T_50%_ (decomposition temperature at 50 wt% weight loss), T_max_ (temperature at maximum thermal decomposition rate), rate at T_max_ (thermal decomposition rate at T_max_), and W_800_ (char residues at 800 °C) are reported in Table 3.

The TGA and DTG thermograms in Figure 3 show that neat PBS had only one thermal decomposition step at around 340–420 °C and a single decomposition peak (T_max_) at around 400 °C. Furthermore, there was almost nothing left at 800 °C. The decomposition patterns of the 30APP/PBS and the 30WHF/PBS composites were different from that of the neat PBS. Their initial decomposition temperature based on 5% weight loss (T_5%_) was lower than that of the neat PBS, while their residual char yield at 800 °C (W_800_) was higher. The reduction in T_5%_ was caused by the lower initial decomposition temperatures of APP and WHF. The 30APP/PBS composite had two decomposition peaks at around 350 °C and 615 °C. The first decomposition peak (T_max1_) was attributed to the premature decomposition of APP, which produced phosphoric acid [33]. The acid accelerated the PBS decomposition and led to the formation of char, as evidenced by a weight plateau between 350 and 550 °C. The second decomposition peak (T_max2_) was caused by the evaporation and/or dehydration of phosphoric acid to form P_2_O_5_ and P_4_O_10_, as well as the decomposition of the previously formed char [42]. The DTG thermogram of the 30WHF/PBS composite exhibited an additional peak between 320 and 370 °C, corresponding to cellulose decomposition [43]. As summarized in Table 3, the W_800_ of the 30WHF/PBS composite was 2.7 times higher than that of the 30APP/PBS composite. The results suggested that APP was less effective than WHF in producing a char layer for PBS. Nonetheless, the addition of a single filler, either WHF or APP, to PBS was still inadequate to improve its char-forming abilities.

All WHF/APP/PBS composites, with the exception of the 9WHF/36APP/PBS composite, had a one-step decomposition, as shown in Figure 3b. The T_5%_, T_50%_, and T_max_ values of all WHF/APP/PBS composites were lower than neat PBS, while their W_800_ values were higher. The increase in char residue at elevated temperatures was a positive signal that reflected the enhanced flame retardancy of the PBS composites [44].

When the overall IFR weight was held at 45%, T_50%_, T_max_, and W_800_ values of the WHF/APP/PBS composites increased, whereas their T_5%_ and rate of thermal decomposition at T_max_ decreased as the WHF:APP weight ratio varied from 1:4 to 1:2. Among these, the 15WHF/30APP/PBS composite (WHF:APP ratio of 1:2) had the lowest T_5%_ and rate of thermal decomposition at T_max_, while its W_800_ was the highest. As mentioned earlier, APP and WHF decomposed at temperatures lower than PBS. When the hydroxyl groups of WHF came into contact with the phosphoric acid molecules produced by APP decomposition, a protective char layer formed. Nonetheless, the excess APP in the 9WHF/36APP/PBS composite (a WHF:APP ratio of 1:4) can also interact with the PBS matrix to generate char [20,24,31]. As this char proved unstable at elevated temperatures, the thermogram of the 9WHF/36APP/PBS composite revealed a two-step decomposition.

When the ratio of WHF to APP was 1:2 and the overall IFR amount decreased from 45% to 30%, the W_800_ of the WHF/APP/PBS composites decreased from 20.93% to 7.53%, while the rate of thermal decomposition at T_max_ increased. The 10WHF/20APP/PBS composite containing 30% IFR had the highest T_5%_ and rate of thermal decomposition at T_max_ but the lowest W_800_.These results implied that when the overall IFR amount was less than 45%, there was insufficient IFR content to form char residue.

The TGA results suggested that the thermal stability and char-forming abilities of PBS were significantly improved by the addition of WHF and APP in combination. These abilities were also influenced by the ratio of WHF to APP, as well as the total amount of IFR. To assess the impacts of IFR components, i.e., WHF and APP, on PBS combustion behavior, 15WHF/30APP/PBS and 10WHF/20APP/PBS composites were further investigated in the following section.

### 3.4. Combustion Behavior of WHF/APP/PBS Composites

**Figure 4:**
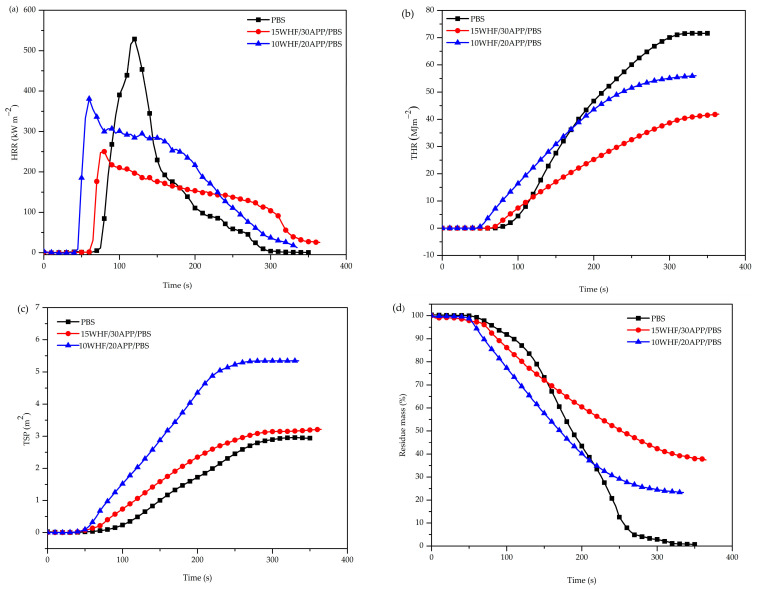
Combustion behaviors of neat PBS and WHF/APP/PBS composites as functions of combustion time at a heat flux of 35 kW/m^2^; heat release rate (**a**), total heat release (**b**), total smoke production (**c**), and residue mass (**d**).

In this study, the impacts of WHF and APP on the combustion behaviors of neat PBS and PBS composites, 15WHF/30 APP/PBS and 10WHF/20APP/PBS, were evaluated using a cone calorimeter. The combustion behaviors of neat PBS and WHF/APP/PBS composites, i.e., heat release rate (HRR), total heat release (THR), total smoke production (TSP), and residue mass were determined as functions of combustion time, with results illustrated in Figure 4a–d. Critical parameters, including time-to-ignition (TTI), peak heat release rate (pHRR), time-to-peak heat release (t_pHRR_), fire performance index (FPI), fire growth rate index (FIG), flame retardancy index (FRI), total heat release (THR), total smoke production (TSP), and residue mass are summarized in Table 4.

TTI refers to the point at which the concentration of combustible gases released during the material’s decomposition reaches a level that supports ignition. As illustrated in Figure 4 and reported in Table 4, the TTI of neat PBS was 75 s. Both 15WHF/30APP/PBS and 10WHF/20APP/PBS composites showed a reduction in TTI to 65 and 45 s, respectively. The relatively shortened TTI values of the WHF/APP/PBS composites were attributed to the early decomposition of WHF and APP, as evidenced by the TGA results.

HRR and pHRR play essential roles in understanding the initiation, spread, and intensity of fires in actual fire scenarios. pHRR is proportional to the maximum magnitude of HRR, and is related to fire development and flashover incidents. Figure 4a depicts HRR curves of PBS and WHF/APP/PBS composites. Neat PBS burned quickly after ignition and had a sharp pHHR at 120 s, with a value of 528.59 kW/m^2^. Despite the fact that both of WHF/APP/PBS composites exhibited shorter TTI values than neat PBS, the WHF/APP/PBS composites exhibited substantially milder burning tendencies. The pHRR values of 15WHF30/APP/PBS and 10WHF/20APP/PBS composites reduced drastically to 250.10 and 380.93 kW/m^2^, respectively, representing reductions of 52% and 28% compared to neat PBS. This indicated that addition of WHF and APP at weight ratio of 1:2 improved the flame-retardant performance of WHF/APP/PBS composites in terms of preventing the composites from burning violently and suppressing the flames from spreading.

The value of THR was used to evaluate the fire safety of the materials in real life, while the slope of the THR curve reflected the spread of the fire and illustrated the fire extension. The THR curves of neat PBS and WHF/APP/PBS composites are illustrated in Figure 4b. Neat PBS possessed a higher ultimate THR value and a steeper THR curve than the WHF/APP/PBS composites. At the end of combustion, neat PBS released a total heat of 71.7 MJ/m^2^, while the 15WHF/30APP/PBS and 10WHF/20APP/PBS composites had THR values of 41.8 and 56.0 MJ/m^2^, respectively. In addition, the 15WHF/30APP/PBS composite had a lower THR value and flatter THR curve than the 10WHF/20APP/PBS composite. This indicated that the 15WHF/30APP/PBS composite restrained the heat release of the PBS matrix more effectively than the 10WHF/20APP/PBS composite.

The fire performance index (FPI) and fire growth rate index (FIG) are important parameters for evaluating the danger of a fire in actual fire situations and gaining insight into the effectiveness of flame retardants. The FPI is defined as the ratio of TTI to pHRR, as shown in Equation (1). The FPI value is frequently used to predict whether a material would produce rapid, intense combustion after ignition. In general, fire resistance improves as FPI values increase. The FIG is a universal index for estimating the magnitude and spread rate of a fire. It is the ratio between pHRR and the t_pHRR_, as shown in Equation (2). Flame propagation and expansion will occur more rapidly as the FIG value increases [14].

(1) $$\mathrm{Fire}\;\mathrm{performance}\;\mathrm{index}\;(\mathrm{FPI})=\frac{TTI}{pHRR}$$

(2) $$\mathrm{Fire}\;\mathrm{growth}\;\mathrm{rate}\;\mathrm{index}\;(\mathrm{FIG})=\frac{pHRR}{t_{pHRR}}$$

As shown in Table 4, the FPI value of the 10WHF/20APP/PBS composite with a total IFR content of 30 wt% was equivalent to neat PBS; however, its FGI value was higher. With a total IFR content of 45 wt%, the FPI of the 15WHF/30APP/PBS composite (0.26 m^2^s/kW) was higher than that of the neat PBS (0.14 m^2^s/kW). Furthermore, the FIG of the 15WHF/30APP/PBS composite decreased to 3.33 kW/m^2^s compared to that of the neat PBS (4.40 kW/m^2^s). This indicated that the flame retardancy of the 15WHF/30APP/PBS composite was superior to that of the neat PBS by suppressing flame growth and decreasing material combustion intensity. These results suggested that the amount of IFR and the WHF:APP ratio are crucial parameters for enhancing the flame-retardant performance of PBS composites. In this study, the addition of WHF and APP at a weight ratio of 1:2 and total IFR content of 45 wt% considerably enhanced the fire safety performance of PBS composites.

Additionally, the flame retardancy index (FRI), developed by Vahabi et al., offers a method for quantifying the flame-retardant performance of thermoplastic composites [45,46]. Equation (3) can be employed to determine the FRI value of a given material. A value of FRI greater than 1 signifies a “good” flame retardancy, indicating an enhanced level of thermal stability. As the FRI value increases, the flame-retardant performance improves accordingly.

(3) $$\mathrm{Flame}\;\mathrm{retardancy}\;\mathrm{index}\;(\mathrm{FRI})=\frac{{\left[THR\times \left(\frac{pHRR}{TTI}\right)\right]}_{neatpolymer}}{{\left[THR\times \left(\frac{pHRR}{TTI}\right)\right]}_{composite}}$$

The values of the FRI for the WHF/APP/PBS composites are summarized in Table 4. Both the 15WHF/30APP/PBS and 10WHF/20APP/PBS composites had FRI values greater than 1, indicating that they both had higher levels of flame retardancy than the neat PBS. The FRI value of the 15WHF/30APP/PBS composite was, however, greater than that of the 10WHF/20APP/PBS composite. This suggested that the 15WHF/30APP/PBS composite provided superior flame-retardant properties.

When a fire occurs, the death rate caused by smoke is greater than that caused by flames. The smoke generated during the fire reduces the visibility of the fire hazard site, while people trapped inside have less chance of escaping if they breathe in hazardous gases. Thus, the total smoke production (TSP) of materials during combustion is a signi ficant focus parameter for evaluating the fire hazard of a flame-retarded material. Figure 4c shows TSP curves of neat PBS and WHF/APP/PBS composites. PBS had a TSP value of only 2.9 m^2^, indicating that it was a flammable but not smoky polymeric material. After the addition of WHF and APP at a total filler of 30 wt%, the TSP value of 10WHF/20APP/PBS composite was higher than that of the neat PBS due to the incomplete combustion and smoke production of the IFR system. During the decomposition of APP, phosphoric acid is produced, along with emission of solid particles and non-combustible gases such as vaporous water, carbon dioxide, ammonia, and others [47]. Simultaneously, the phosphoric acid formed during APP decomposition reacts with the hydroxyl group of WHF, resulting in the formation of intumescent char, which then releases cyclic compounds [33]. A significant emission of volatile gases also occurs due to the combustion of the PBS matrix and WHF. The escape of volatile gases pushes out unstable carbon particles, non-combustible gases, and cyclic compounds, resulting in considerably increased total smoke production [33]. However, with the addition of WHF and APP at a total filler content of 45 wt%, the TSP value of the 15WHF/30APP/PBS composite was 3.0 m^2^ and comparable to that of the neat PBS. The results indicated that the WHF and APP formulation with a weight ratio of 1:2 and total IFR content of 45 wt% was optimal. Both components reacted favorably during combustion to make the char layer dense and compact, as evidenced by SEM micrographs of char residues of the WHF/APP/PBS composites after the cone calorimetry test. The generated char prevented the escape of combustible gases and smoke precursors and contributed to the reduction in smoke emissions.

The mass loss rate reflects the charring effect of a material during combustion. A slow mass loss rate indicates the stability of the char layer, while high char residue corresponds with high char formation. The results in Figure 4d show that the mass of neat PBS significantly reduced after ignition. Neat PBS had almost completely combusted after 300 s, with only 0.73% char residue remaining. After adding WHF and APP at a weight ratio of 1:2, the mass loss rate of WHF/APP/PBS composites was slower than that of the neat PBS; however, their char residues were higher. After combustion, the char residues in 15WHF/30APP/PBS and 10WHF/20APP/PBS composites were 37.42% and 23.30%, respectively. The produced char layer acted as a physical barrier to protect the PBS matrix from heat and oxygen transfer, resulting in a decrease in pHRR, THR, and TSP values. These were consistent with the TGA results, which showed that neat PBS had no char residue, whereas the 15WHF/30APP/PBS composite exhibited superior char residue in comparison to the 10WHF/20APP/PBS composite. The findings suggested that the optimal weight ratio of WHF:APP and total weight of IFR optimized the formation of a stable carbon layer.

### 3.5. Morphologies and Structure of Char Residue

**Figure 5:**
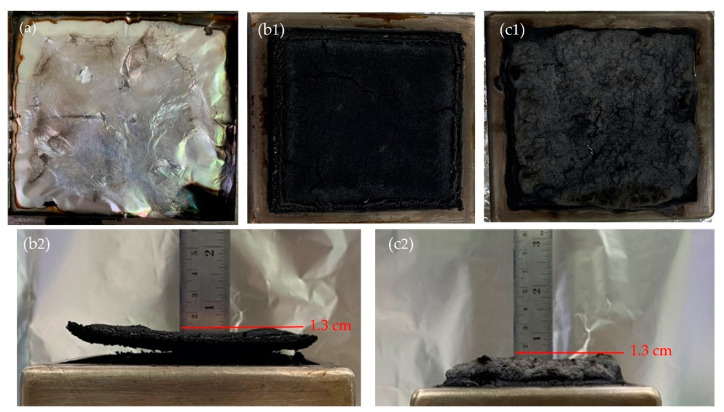
Digital images of residues of neat PBS and WHF/APP/PBS composites after the cone calorimetry test, PBS (**a**), 15WHF/30APP/PBS (**b1**,**b2**), and 10WHF/20APP/PBS (**c1**,**c2**).

Digital images of residues of neat PBS and WHF/APP/PBS composites after the cone calorimetry test are shown in Figure 5. In contrast to the almost nonexistent char residue left behind by the neat PBS in Figure 5a, large amounts of intumescent chars were generated in both the 15WHF/30APP/PBS and 10WHF/20APP/PBS composites after combustion, as illustrated in Figure 5(b1–c2). The side views of char residues of WHF/APP/PBS composites in Figure 5(b2,c2) reveal that the char layer in the WHF/APP/PBS composites was around 1–1.5 cm thick. The specimen’s thickness increased by 233–400% from 3 mm before burning, indicating that intumescent char was produced when WHF and APP were added to the PBS matrix at a weight ratio of 1:2.

**Figure 6:**
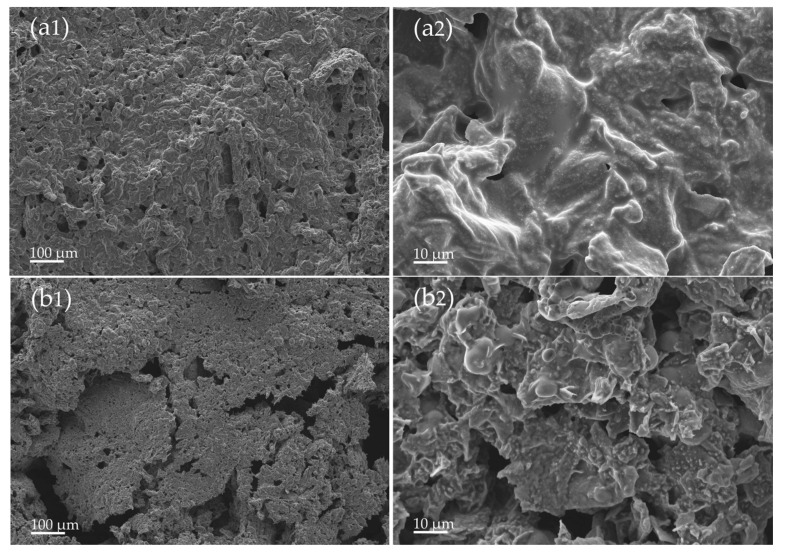
SEM micrographs of the surface char layer of WHF/APP/PBS composites with 100× (1) and 1.00k× (2) magnifications after the cone calorimetry test: 15WHF/30APP/PBS (**a1**,**a2**) and 10WHF/20APP/PBS (**b1**,**b2**) composites.

To improve the understanding of the influence of WHF and APP on char formation during combustion, the exterior surfaces of the char residues of WHF/APP/PBS composites after the cone calorimetry test were analyzed using SEM, with their micrographs shown in Figure 6.

As seen in Figure 6(a1,a2), the char layer of the 15WHF/30APP/PBS composite was compact and continuous with few surface holes and fractures. A 1000× magnification revealed that the surface of the dense char layer was wrinkly and convex. The morphologies of the char residues supported previous findings that 15WHF/30APP/PBS composites improved HRR, ultimate THR, and TSP values compared to neat PBS, and were UL-94 V-0 certified without polymer melt-dripping. As Yu et al. also observed and reported, the compact carbon layer was effective in isolating oxygen and heat transmission, whereas wrinkled and convex structures were superior in blocking oxygen and preventing the escape of hazardous gases [15]. The 10WHF/20APP/PBS composite produced a large expandable char residue, but its char residue in Figure 6(b1,b2) was thin, fragile, and had several surface defects, including sizable holes and collapses. As illustrated in Figure 4, the 10WHF/20APP/PBS composite had the shortest time to ignition (TTI) and time to pHRR, as well as the highest total heat release at initial combustion time (<200 s). These may be the reasons for the 10WHF/20APP/PBS composite’s weak, unstable char residue. The char was incapable of withstanding the impact of the massive amount of gas released rapidly during combustion. The evidence confirmed why the 10WHF/20APP/PBS composite had higher TSP value compared to neat PBS, as well as a UL-94 V-2 rating with significant polymer melt-dripping.

**Figure 7:**
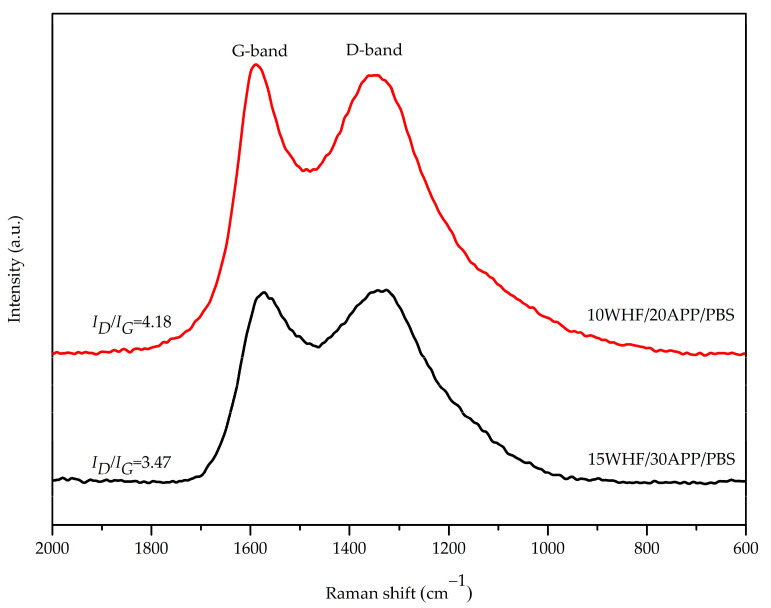
FT-Raman spectra of char residues of 15WHF/30APP/PBS and 10WHF/20APP/PBS composites after the cone calorimetry test.

The degree of graphitization of the char residues of WHF/APP/PBS composites was evaluated using FT-Raman spectroscopy. Raman spectra of the char residues of 15WHF/30APP/PBS and 10WHF/20APP/PBS composites are shown in Figure 7. Their spectra revealed two distinct peaks at around 1600 cm^−1^ and 1350 cm^−1^, which were assigned to the G peak and the D peak, respectively. Generally, the G peak is related to the vibration of sp^2^-hybrided carbon atoms of the crystalline graphite layer, whereas the D peak is associated with the vibration of sp^3^-hybrided carbon atoms of disordered graphite or amorphous carbon [27,48]. The results indicated that graphitic carbon and disordered carbon structures coexisted in the char residues of 15WHF/30APP/PBS and 10WHF/20APP/PBS composites.

The degree of graphitization of a char residue is inversely proportional to the ratio of the integrated areas of the D and G peaks (I_D_/I_G_). When the degree of graphitization increases, the char residue becomes more resistant to thermal oxidation. This is because graphitic carbon is more thermally resistant than its amorphous form. Hence, a highly gra-phitized char residue may function as a stable and effective thermal barrier, thereby enhancing the flame-retardant properties of a material [49,50]. In order to quantify the degree of graphitization of the char residues in PBS composites, their D and G peaks in Figure 7 were resolved and then integrated using Gaussian fitting. The I_D_/I_G_ ratios for the char residues of 15WHF/30APP/PBS and 10WHF/20APP/PBS composites were 3.47 and 4.18, res-pectively. As a result, the char residue of the 15WHF/30APP/PBS composite was more thermally stable and stronger than that of the 10WHF/20APP/PBS composite. This result supported why the 15WHF/30APP/PBS composite exhibited superior intumescent flame-retardant properties compared to the 10WHF/20APP/PBS composite.

### 3.6. Evolved Products during Thermal Decomposition of WHF/APP/PBS Composites

**Figure 8:**
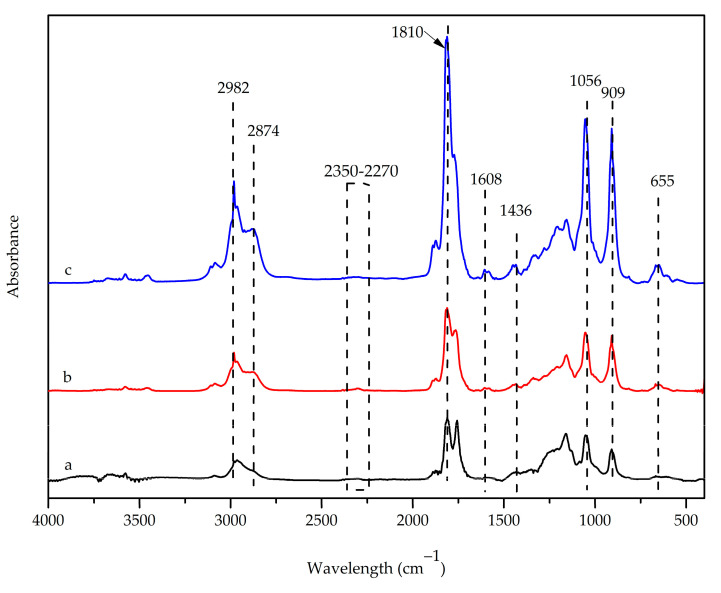
FTIR spectra of the gaseous products generated at T_max_ of neat PBS (a), 15WHF/30APP/PBS (b), and 10WHF/20APP/PBS (c) composites.

**Figure 9:**
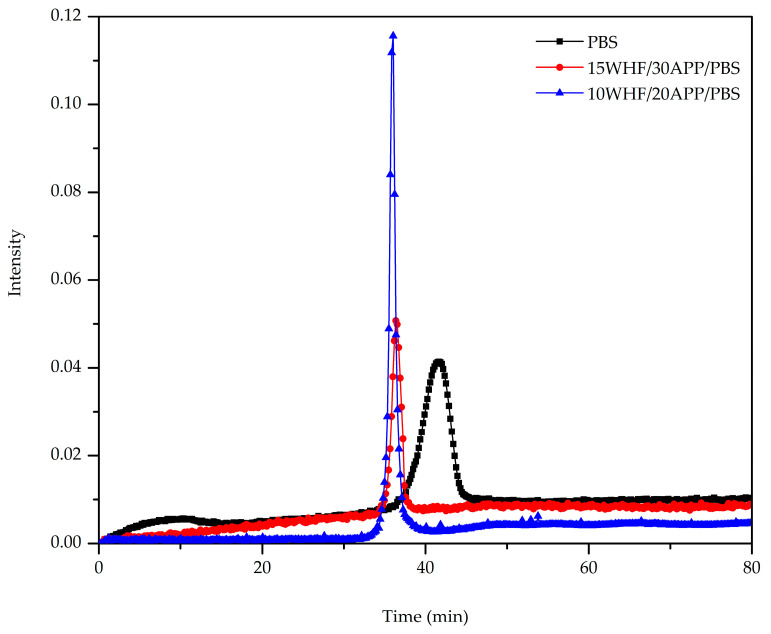
Intensity of the gaseous products generated during decomposition of neat PBS, 15WHF/30APP/PBS, and 10WHF/20APP/PBS composites as a function of time.

Gaseous products generated at the T_max_ of neat PBS and WHF/APP/PBS composites were monitored and analyzed by a Fourier transform infrared spectrometer that was directly coupled to a thermal gravimetric analyzer. The area under a spectrum corres- ponded to total infrared intensity. Their FTIR spectra and absorption intensities are depicted in Figure 8 and Figure 9, respectively.

The FTIR spectrum of the gaseous products generated at the T_max_ of neat PBS exhi- bited absorption peaks at 2982 and 2874 cm^−1^, which were attributed to C-H stretching of the -CH_2_ and -CH_3_ groups, respectively. The peak at around 1436 cm^−1^ was related to -CH_3_ bending, with bands at 1810 cm^−1^ (C=O stretching) and 1056 cm^−1^ (C-O-C stretching) related to the presence of succinic anhydride and aliphatic ether. The peak at 909 cm^−1^ was due to the absorption of vinyl C-H bending of gaseous alkene [22,51]. The bands appearing at 2350–2270 cm^−1^ (asymmetric stretching of O=C=O) and 655 cm^−1^ (bending of O=C=O) were related to the formation of carbon dioxide [52]. The identified functional groups and earlier studies suggested that the primary gaseous products from PBS decomposition were alkene, succinic acid, butanediol, succinic anhydride, aliphatic ether, and carbon dioxide.

FTIR spectra of volatile products generated at T_max_ of the 15WHF/30APP/PBS and 10WHF/20APP/PBS exhibited an additional peak at 1608 cm^−1^ (C=C-C aromatic ring stretching), which was attributed to lignin in WHF [53]. The intensity of the peak at 909 cm^−1^ increased considerably during the decomposition of the WHF/APP/PBS composites. This occurred because of the overlap between the absorption of the C-H deformation vibration of gaseous alkenes and the absorption peak of ammonia gas produced during the decomposition of APP [22]. The increased intensity of the O=C=O peak at 655 cm^−1^ was also attributed to the release of carbon dioxide during the decomposition of WHF, which is rich in carbon atoms and hydroxyl groups [54].

Figure 9 shows the intensity of gaseous products from the decomposition of neat PBS and WHF/APP/PBS composites as a function of time. The WHF/APP/PBS composites reached their maximum rate of gaseous product generation earlier than neat PBS. In addition, the 15WHF/20APP/PBS composite produced a similar amount of gaseous products as neat PBS, since the absorbance intensities of their generated gases were comparable. In comparison, neat PBS and the 15WHF/30APP/PBS composite emitted lower amounts of evolved gases than the 10WHF/20APP/PBS composite. The result validated the char residue morphologies and FT-Raman results, where the decomposition of the 15WHF/30APP/PBS composites produced a dense char layer with a high graphitization degree, which effectively prevented the volatile products from escaping.

### 3.7. Proposed Intumescent Flame-Retardant Mechanisms

**Figure 10:**
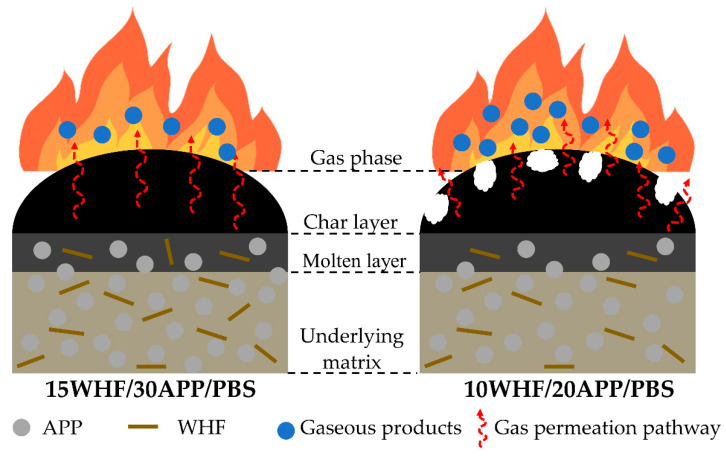
Proposed intumescent flame-retardant mechanisms for 15WHF/30APP/PBS and 10WHF/20APP/PBS composites.

The flame-retardant performance of PBS was enhanced by employing an intumescent system consisting of APP and WHF. The ratio of WHF to APP and the IFR content were found to significantly influence the flame-retardant efficacy of WHF/APP/PBS composites. All WHF/APP/PBS composites exhibited intumescent char after combustion, but the PBS composite with enhanced flame retardancy was only produced utilizing the optimal WHF to APP ratio and total IFR content. In comparison to other PBS composites with the same total IFR content (45 wt%), the 15WHF/30APP/PBS composite with WHF:APP ratio 1:2 had superior flame-retardant properties and yielded the highest char residue amount in the TGA test. When setting the WHF: APP ratio to 1:2, the 10WHF/20APP/PBS composite with an IFR content of 30% showed the lowest flame-retardant properties and provided the lowest char residue content in the TGA test. Differences in flame-retardant performance between the 15WHF/30APP/PBS and 10WHF/20APP/PBS composites were investigated, with the proposed intumescent char-forming mechanisms illustrated in Figure 10.

When the WHF/APP/PBS composites were heated, APP thermally decomposed into phosphoric acid, water and ammonia. The released incombustible ammonia gas diluted the oxygen concentration in the combustion zone, slowing the combustion and decreasing heat release. Cross-linking between the released phosphoric acid molecules generated po-lyphosphoric acid and water. Then, polyphosphoric acid reacted with the hydroxyl group of WHF to form a protective char layer, while also catalyzing the decomposition of the PBS matrix to release small molecules such as carbon dioxide. The accumulation of incombustible gases like ammonia, as well as small olefin/alkane molecules, carbon dioxide, and water, promoted the expansion of the char layer [14,15,20]. The intumescent char layer of the 10WHF/20APP/PBS composite was unstable and insufficiently strong to prevent the massive volatile gases from escaping. Therefore, the penetration of the released gases caused numerous cracks and holes on its surface. This resulted in decreased flame-retardant performance and the high total smoke release (TSP) of the 10WHF/20APP/PBS composite. In contrast, the char layer of the 15WHF/30APP/PBS composite was coherent and dense, with a high degree of graphitization. This char acted as a stable barrier, reducing the release of flammable gases and heat, slowing down the combustion process and preventing melt dripping of PBS.

### 3.8. Tensile Properties of PBS and PBS Composites

**Figure 11:**
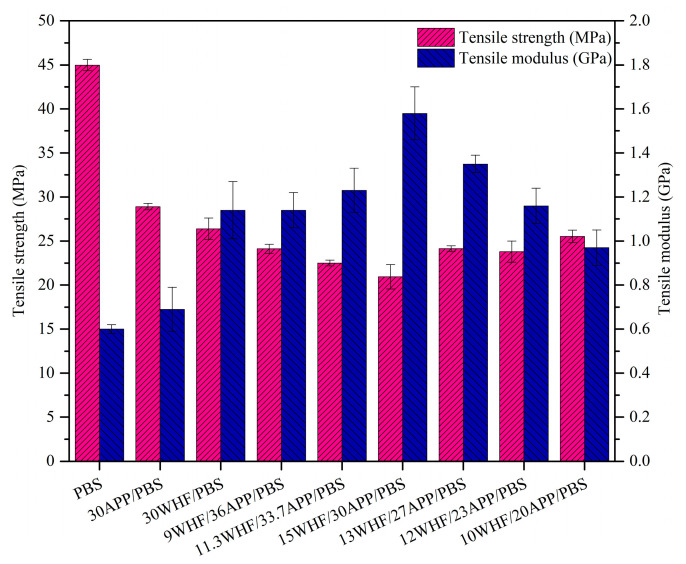
Tensile properties of neat PBS and PBS composites.

Tensile properties of PBS and its composites are shown in Figure 11. It is clear that the addition of WHF and/or APP has a considerable impact on the tensile properties of PBS. The tensile strength of the APP/PBS, WHF/PBS, and WHF/APP/PBS composites was obviously lower than that of neat PBS, but their tensile modulus was much higher. The 10WHF/20APP/PBS composite had the highest tensile strength among the PBS composites, with a value of 25.52 MPa, which was 43% lower than that of neat PBS. The deterioration of the tensile strength of PBS could be attributed to poor compatibility between the hydrophilic WHF-APP fillers and the hydrophobic PBS matrix. A portion of the additives partially agglomerated in the PBS matrix, and the stress concentration formed around the agglomerated particles, lowering the mechanical performance of the PBS [5]. Nonetheless, the tensile modulus of the 15WHF/30APP/PBS composite reached 1.58 GPa, an increase of 163% compared to that of neat PBS.

### 3.9. Comprehensive Performance of WHF/APP/PBS Composites

**Figure 12:**
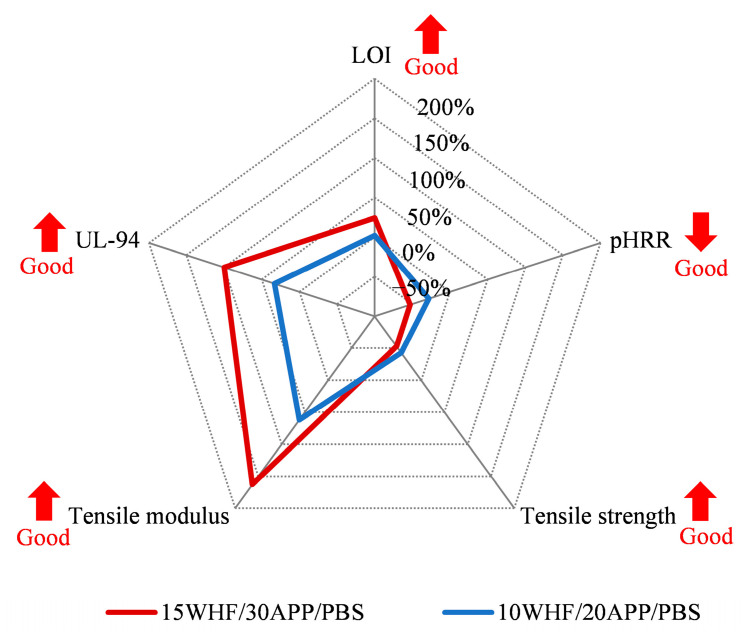
Comprehensive performance comparison between 15WHF/30APP/PBS and 10WHF/20APP/PBS composites.

The influence of WHF and APP on the overall performance of PBS is depicted graphi-cally in Figure 12. Incorporating WHF and APP improved the flame-retardant characteristics of PBS, as seen by the simultaneous rise of LOI and UL-94 and a decrease in pHRR. In comparison, the 15WHF/30APP/PBS composite exhibited better flame-retardant pro-perties than the 10WHF/20APP/PBS composite. In terms of tensile properties, both the 15WHF/30APP/PBS and 10WHF/20APP/PBS composites exhibited a noticeable decrease in tensile strength and a significant increase in tensile modulus when compared to neat PBS. Consequently, the combined utilization of WHF and APP as intumescent flame retardants presented a potential avenue for developing bio-based flame-retardant polymer composites suitable for engineering applications. Nonetheless, further improvement is necessary to enhance the mechanical properties in future endeavors.

## 4. Conclusions

This study incorporated WHF and APP to create an intumescent flame-retardant system for PBS composites. TGA analysis revealed the improved char yields and thermal stability of the PBS composites compared to those of neat PBS. Notably, the 15WHF/30APP/PBS composite demonstrated a notable LOI value of 28.8% and met the UL-94 V-0 rating without dripping. Moreover, the composite exhibited reduced pHRR and THR compared to the neat PBS. The resulting char residue displayed a highly graphitized structure with a dense, smooth surface, indicating enhanced fire resistance. Al-though there was a decrease in tensile strength, there was a significant increase in tensile modulus for both the 15WHF/30APP/PBS and 10WHF/20APP/PBS composites compared to that of neat PBS. This suggests the potential for the development of an eco-friendly flame-retardant system utilizing WHF and APP for PBS composites. By optimizing the ratio of WHF to APP at the appropriate IFR content, the flame-retardant properties of WHF/APP/PBS composites can be further improved. Despite the need for enhancements in mechanical properties, the application potential of these PBS composites in fields such as electronic devices, construction, and transportation appears promising.

## Figures and Tables

**Table 1:** Designations and formulations of WHF/APP/PBS composites.

Sample	WHF:APP Ratio	Total IFR (wt%)	PBS (wt%)	PBS-*g*-GMA (wt%)	WHF (wt%)	APP (wt%)
PBS	-	-	100	-	-	-
30APP/PBS	-	30	65	5	-	30
30WHF/PBS	-	30	65	5	30	-
9WHF/36APP/PBS	1:4	45	50	5	9	36
11.3WHF/33.7APP/PBS	1:3	45	50	5	11.3	33.7
15WHF/30APP/PBS	1:2	45	50	5	15	30
13WHF/27APP/PBS	1:2	40	55	5	13.3	26.7
12WHF/23APP/PBS	1:2	35	60	5	11.7	23.3
10WHF/20APP/PBS	1:2	30	65	5	10	20

**Table 2:** LOI and UL-94 performance of PBS and PBS composites.

Sample			LOI (%)	UL-94				Horizontal Burning Rate (mm/min)
	WHF: APP Ratio	Total IFR Content (wt%)		t_1_ (s)	Dripping	Ignite the Absorbent Cotton	Rating	
PBS	-	-	23.3	>30	Yes	Yes	NC	16.39
30APP/PBS	-	30	32.0	-	Yes	No	V-0	-
30WHF/PBS	-	30	20.6	>30	Yes	Yes	NC	14.45
9WHF/36APP/PBS	1:4	45	25.5	4	Yes	No	V-0	-
11.3WHF/33.7APP/PBS	1:3	45	27.4	2	No	No	V-0	-
15WHF/30APP/PBS	1:2	45	28.8	-	No	No	V-0	-
13WHF/27APP/PBS	1:2	40	24.5	28	Yes	Yes	V-2	9.75
12WHF/23APP/PBS	1:2	35	24.4	20	Yes	Yes	V-2	8.25
10WHF/20APP/PBS	1:2	30	23.7	22	Yes	Yes	V-2	3.75

**Table 3:** Thermal decomposition temperatures and weight loss of PBS and PBS composites.

Sample	WHF:APP Ratio	Total IFR Content (wt%)	T_5%_ (°C)	T_50%_ (°C)	T_max1_ (°C)	Rate at T_max_ (%/min)	T_max2_ (°C)	W_800_ (wt%)
PBS	-	-	341.8	395.8	401.8	0.40	-	-
30APP/PBS	-	30	330.2	352.5	352.3	1.77	613.3	4.04
30WHF/PBS	-	30	271.2	391.2	396.3	0.28	-	10.93
9WHF/36APP/PBS	1:4	45	298.8	347.5	347.2	1.53	632.5	7.54
11.3WHF/33.7APP/PBS	1:3	45	280.7	346.8	345.8	1.39	-	13.79
15WHF/30APP/PBS	1:2	45	220.2	351.3	349.7	0.62	-	20.93
13WHF/27APP/PBS	1:2	40	245.7	346.2	345.2	1.34	-	10.98
12WHF/23APP/PBS	1:2	35	249.2	346.0	345.5	1.68	-	9.24
10WHF/20APP/PBS	1:2	30	253.3	344.7	344.8	1.75	-	7.53

**Table 4:** Critical combustion parameters from the cone calorimetry test.

Sample	Time to		pHRR (kW/m^2^)	FPI (m^2^s/kW)	FIG (kW/m^2^s)	FRI	THR (MJ/m^2^)	TSP (m^2^)	Residue (wt%)
	Ignition (s)	pHRR (s)							
PBS	75	120	528.59	0.14	4.40	-	71.7	2.9	0.73
15WHF/30APP/PBS	65	75	250.10	0.26	3.33	3.14	41.8	3.0	37.42
10WHF/20APP/PBS	45	60	380.93	0.13	6.35	1.06	56.0	5.3	23.30

## Data Availability

The data presented in this study are available in the present article.
